# Complete chloroplast genome of *Dactylicapnos torulosa* (Hook. & Thoms.) Hutch., a medicinal plant from southwest China and its phylogeny

**DOI:** 10.1080/23802359.2021.1999868

**Published:** 2021-11-18

**Authors:** Haisu Hu, Dequan Zhang

**Affiliations:** a College of Pharmacy, Dali University, Dali, China; b Yunnan Key Laboratory of Screening and Research on Anti-pathogenic Plant Resources from Western Yunnan, Dali, China

**Keywords:** Chloroplast genome, *Dactylicapnos torulosa* (Hook. & Thoms.) Hutch., Illumina sequencing, Phylogenetic analysis

## Abstract

*Dactylicapnos torulosa* (Hook. & Thoms.) Hutch. is a commonly used medicinal plant in southwest China. In this study, we sequenced the complete chloroplast genome of *D. torulosa* and explored phylogenetic relationship of the species and its related species. The genome size is 166,238 bp with typical quadripartite structure. A pair of inverted repeats (IRs) of 37,115 bp are separated by a small single copy (SSC) region of 18,627 bp and a large single copy (LSC) region of 73,381 bp. In total, 107 genes are annotated, including 76 protein coding genes, 27 transport RNA, four ribosomal RNA. Moreover, 13 genes are found to contain introns. Phylogenetic analysis showed that *Dactylicapnos torulosa* formed a basal branch instead of gathering together with *Corydalis*, which was inconsistent with the traditional treatment on Fumarioideae. This study provides useful information for phylogeny and conservation of the species and its relatives.

*Dactylicapnos* Wall. is a small genus in the family Papaveraceae, which includes 7 species all over the world. There are 4 species in China, nearly distributing in the southwest China. And *D. torulosa* is mainly located in Tibet, Yunnan and Sichuan provinces (Zhang et al. 2008). Whole grass of the species is generally used as folk medicines, which has efficacy of treating cough (Rücker et al. 1994; Cao et al. 2014). Until now, most of the studies on *D. torulosa* focused on biological characteristics, medicinal value, and effective components. However, there is still no report on complete chloroplast genome of *D. torulosa*. Here, we sequenced the complete chloroplast genome of this species and explored phylogeny between *Dactylicapnos* and related genera in Papaveraceae. It would be beneficial to species discrimination, phylogenetic study, and conservation of species in the family.

In this study, molecular material of *D. torulosa* was dried leaf using silica gel. It was collected from Eryuan county in Yunnan province, China (26°05′08.1″N, 99°57′37.62″E) with an altitude of 2079 m. The voucher specimen (No. ZDQ125) was deposited in the Herbarium of Medicinal Plants and Crude Drugs of the college of Pharmacy, Dali University (Professor Dequan Zhang, zhangdeq2008@126.com). Total genomic DNA was extracted from dried leaf by a modified CTAB method (Doyle and Doyle 1987) and sequenced using the Illumina Hiseq 2500 platform with paired end (2 × 300 bp) library (Shendure and Ji 2008). Raw data was filtered using Trimmomatic v.0.32 software with default settings (Bolger et al. 2014). Subsequently, the trimmed reads were assembled into contigs using GetOrganelle.py with the complete chloroplast genome of *Corydalis bungeana* Turcz. (MW538958) as reference (Jin et al. 2020). Finally, the complete chloroplast genome was annotated with Geneious 8.0.2 software (Kearse et al. 2012) and submitted to Genbank (accession number: MZ399803). Moreover, the simple sequence repeats (SSRs) were detected using MISA (Murat et al. 2011). Meanwhile, in order to explore phylogeny of *Dactylicapnos* and its related genera, the chloroplast genome sequences of 20 species belonging to five genera in Papaveraceae were downloaded from NCBI database. The chloroplast genomic sequence of *D. torulosa* was aligned using MAFFT v.7.1 with default settings (Jiao et al. 2012). The nucleotide substitution model was determined by jModelTest v 2.1.7 (Darriba et al. 2012). The phylogenetic tree was constructed by the Maximum Likelihood (ML) method with 1000 bootstrap replicates RAxML v.8.2.4 with two species in Lardizabalaceae as the outgroup (Stantakis 2014).

Total length of the complete chloroplast genome of *D. torulosa* is 166,238 bp, including a pair of inverted repeat regions (IRa and IRb, 37,115 bp), a large single copy region (LSC, 73,381 bp), and a small single copy region (SSC, 18,627 bp). In the genome, 107 genes were annotated, including 76 protein coding, 27 tRNA, and four rRNA. Among them, 10 genes contain an intron (*rpl2*, *ndhB*, *ndhA*, *rnL-UAA*, *rpoC1*, *atpF*, *trnK-UUU*, *trnI-GAU*, *trnA-UGG,* and *trnS-CGA*); meanwhile, three genes (*ycf3*, *clpP*, and *rps12*) have two introns. Moreover, the total GC content is 38.1%. Finally, a total of 99 SSRs with five types were detected in complete chloroplast genome of *D. torulosa*, including 75 mono-nucleotide repeats, 10 di-nucleotide repeats, and 14 other types.

The phylogenetic analysis showed that multiple genera in Papaveroideae (including *Meconopsis* Vig., *Papaver* L., *Hylomecon* Maxim., *Chelidonium* L., and *Macleaya* R. Br.) formed an obvious group with high support rate (100%), which also supported its monophyletic status in traditional taxonomy (Zhang et al. 2008) (Figure 1). However, *D. torulosa* did not gathered into one clade with the species in *Corydalis* but located in the basal position within Papaveraceae, though both *Dactylicaponos* and *Corydalis* were usually treated as genera within Fumarioideae (Zhang et al. 2008; Hoot et al. 2015). The complete genome in present study (MZ399803) is the first one in NCBI database, so its phylogenetic position might need more evidence from chloroplast genomes of other species in *Dactylicaponos*. Consequently, this study revealed possible phylogentic position of *D. torulosa* in the Papaveraceae, which would beneficial to phylogeny and conservation of this species and its relatives.

**Figure 1.**
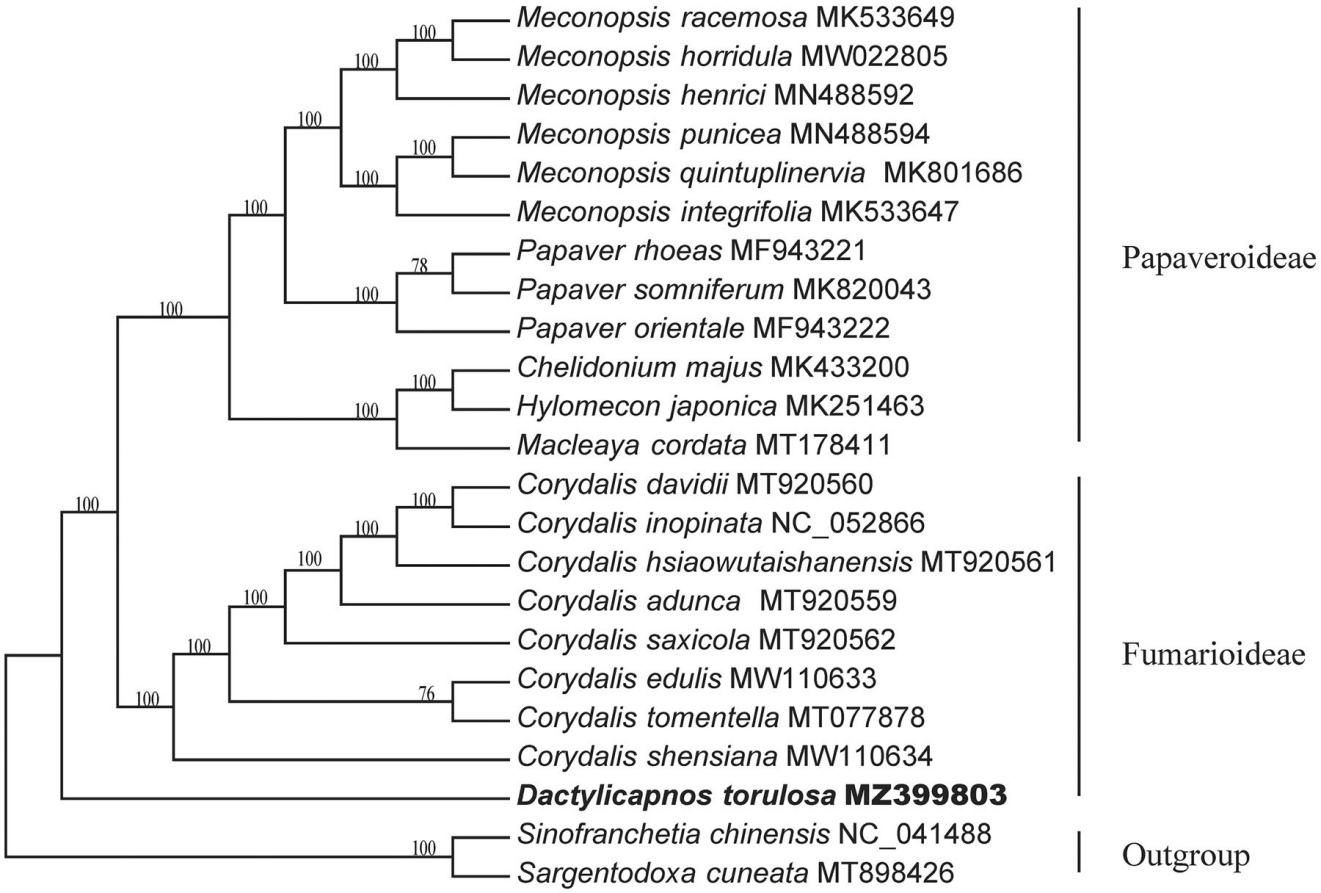
Maximum Likelihood (ML) tree of *D. torulosa* and its related species from the family Papaveraceae. *Sinofranchetia chinensis* and *S. cuneata* from Lardizabalaceae were selected as the outgroup. (Note: numbers beside each node refer to bootstrap values as well as posterior probability).

## Data Availability

The genome sequence data that support the findings of this study are openly available in Genbank of NCBI at (https://www.ncbi.nlm.nih.gov/) under the accession no.MZ399803. The associated BioProject, SRA, and BioSample numbers are PRJNA737567, SRR14826255, and SAMN19700011 repectively.
